# Highly variable antigenic site located at the apex of GII.4 norovirus capsid protein induces cross-reactive blocking antibodies in a variant-specific manner

**DOI:** 10.1128/jvi.00652-25

**Published:** 2025-05-30

**Authors:** Michael Landivar, Kentaro Tohma, Kelsey A. Pilewski, Lauren A. Ford-Siltz, Joseph Kendra, Yamei Gao, Gabriel I. Parra

**Affiliations:** 1 Division of Viral Products, Center for Biologics Evaluation and Research, Food and Drug Administration 333555, Silver Spring, Maryland, USA; University of Michigan Medical School, Ann Arbor, Michigan, USA

**Keywords:** norovirus, calicivirus, cross-reactivity, antibody, diarrhea

## Abstract

GII.4 norovirus is responsible for most cases of viral gastroenteritis worldwide. The complex epidemiology and extreme GII.4 antigenic diversity could challenge the development of a vaccine. Most antibody responses to GII.4 norovirus target five antigenic sites mapping to the major viral capsid, whose variability has been associated with the emergence of antigenically distinct variants. Bioinformatics analyses of amino acid sequences from these antigenic sites demonstrated that antigenic site A is the most variable and relevant site for antigenic diversification. Despite this complex diversity, cross-blocking was demonstrated in the profiling of site A-mapping mouse monoclonal antibodies generated against two antigenically distinct GII.4 variants. Using a novel immunoassay, we determined the contribution of specific epitopes to blockade function in polyclonal sera and observed extensive cross-blocking in which 36% was attributed to antibodies mapping to antigenic site A. However, cross-reactivity was virus-dependent, with the greatest breadth exhibited by contemporary variants that emerged after 2006. Notably, cross-blocking titers associated with antibodies mapping to antigenic site A are differentially affected by an increasing number of mutations on the capsid. Since the elicitation of blocking antibodies to conserved regions of the GII.4 norovirus capsid is infrequent, a better understanding of how to elicit cross-protective responses to immunodominant variable antigenic sites could guide norovirus vaccine development.

**IMPORTANCE:**

GII.4 noroviruses exhibit an accumulation of mutations on their capsid protein, leading to the continuous emergence and turnover of new variants that can escape herd immunity. Despite the fact that most antibodies mapping to the variable antigenic sites of GII.4 norovirus show exquisite specificity, cross-neutralizing antibodies mapping to these variable sites have also been described. In this study, we systematically evaluate the antigenicity of a panel of different GII.4 antigens to demonstrate that cross-reactive responses are elicited in a virus-dependent manner in naïve mice. Notably, one wild-type virus demonstrated multiple instances of potent cross-blocking responses, providing new hopes for the development of cross-protective vaccines against human norovirus.

## INTRODUCTION

Human noroviruses are a main cause of acute gastroenteritis worldwide. While norovirus-induced gastroenteritis is self-limited in most individuals, severe disease can occur in young children, the elderly, and the immunocompromised (1). Although it is widely accepted that a vaccine would be the best strategy to control norovirus disease, the development of such a vaccine has been hindered in large part by our incomplete understanding of immune protection and the extreme genetic and antigenic diversity presented by norovirus (2). Protective antibody responses to human norovirus are mostly elicited to the major capsid protein, VP1 (3, 4). The VP1 presents two structural domains: the shell (S) domain and the protruding (P) domain. The P domain can be further subdivided into two subdomains, P1 and P2 (5). Most variable antigenic sites and the binding sites of histo-blood group antigens (HBGAs) are located on the P2 subdomain (2, 6–9). HBGAs are a large group of carbohydrates that are expressed on the surface of cells and act as attachment factors that facilitate norovirus infection (10–12). Upon heterologous expression, VP1 self-assembles into virus-like particles (VLPs) that morphologically and antigenically resemble wild-type virions (13). Due to the lack of a traditional cell culture system that would allow the amplification of human noroviruses, these VLPs are used as an alternative to study norovirus immune responses and vaccine design. For example, using VLPs, antibody-mediated blockade of the interaction of VP1 with HBGAs has been shown to correlate with protection and virus neutralization (2, 14–16).

**Fig 1:**
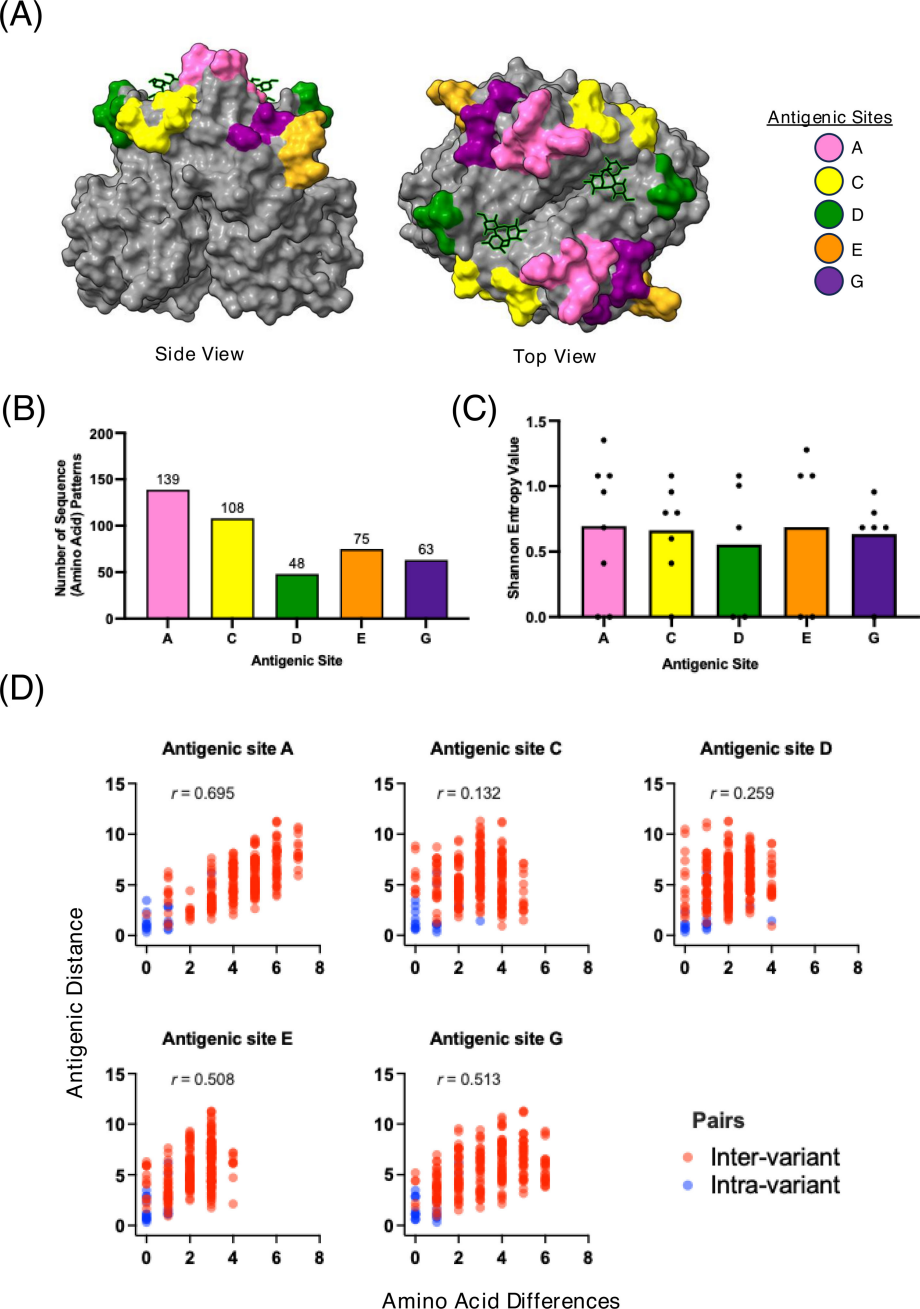
GII.4 norovirus presents large genetic diversification of the major antigenic sites. (**A**) Structural model of GII.4 P domain highlighting the antigenic sites: A, C, D, E, and G. HBGA binding highlighted in lime green and black chain in structural relation to the antigenic sites. (**B**) Count of the number of amino acid sequence patterns of each antigenic site. Colors of bars match legend in (A). (**C**) Average Shannon entropy values of antigenic sites. Colors of bars match sites from legend in (A). (**D**) Spearman’s correlation pairwise analysis of intervariant and intravariant antigenic distances and amino acid differences in respect to major antigenic sites (A, C, D, E, and G). Scatterplot analysis that compares the antigenic distance and amino acid differences on the sequences of each of the major antigenic sites for representative GII.4 VLP pairs. Antigenic distances were derived from Kendra et al. (21). Virus pairs (represented as VLPs) are color-coded to show intervariant (red) and intravariant (blue pairs). Spearman’s coefficient (*r*) measures the strength of association between two variables (33).

Norovirus genotypes are defined based on VP1 genetic differences. Humans can be infected with over 40 genotypes, with the GII.4 genotype responsible for most infections worldwide (17). While the precise mechanism of predominance is poorly understood, the prevailing dogma is that contemporary viruses will be succeeded by viruses associated with a new phylogenetic cluster (namely, variants). These new variants present amino acid substitutions on antigenic sites that allow escape from immunity elicited against previous variants (6, 18–23). To date, several GII.4 variants have been described, but seven have been reported to cause global-scale outbreaks: Grimsby_1995, Farmington Hills_2002, Hunter_2004, Yerseke_2006, Den Haag_2006, New Orleans_2009, and Sydney_2012 (6, 17, 24). The antigenic differences among these variants have been mapped to five major antigenic sites (A, C, D, E, and G) (Fig. 1A), which are located near the HBGA-binding sites (6, 22, 25). Although these variants were shown to be antigenically different (18, 21), varying degrees of cross-blocking have been described at the polyclonal and monoclonal antibody levels (3, 9, 15, 21, 26–28). These cross-blocking responses have been mostly associated with antibodies mapping to the conserved region of the P1 subdomain (29–31), which seem to be less immunogenic than the variable antigenic sites (32).

In this study, we utilized a large panel of mouse sera and monoclonal antibodies (mAbs) developed against GII.4 noroviruses to evaluate the cross-blocking potential of antibodies mapping to the immunodominant antigenic site A. Despite the extensive sequence variability, we observed that cross-blocking antibody responses targeting antigenic site A can be elicited at the monoclonal and polyclonal levels. This cross-reactivity was virus-dependent and did not correlate with amino acid sequence differences. Together, this study provides new insights into immunization strategies to elicit cross-blocking responses to ever-changing GII.4 noroviruses.

## RESULTS

### Antigenic site A is the most variable antigenic site and strongly influences variant antigenic diversification

To determine the role of each antigenic site on GII.4 norovirus antigenic variability, a total of 3,104 VP1 sequences were used to calculate the number of sequence patterns for each of the five antigenic sites in GII.4 viruses circulating worldwide from 1995 to 2022 (Fig. 1B) (25). Antigenic site A showed the most amino acid sequence patterns (*n* = 139) among all the antigenic sites, followed by antigenic site C (*n* = 108) and antigenic site E (*n* = 75) (Fig. 1B). Upon analyzing the entropy of the residues that comprise each antigenic site, site A demonstrated the highest average entropy value (*n* = 0.695), followed by site E (*n* = 0.687) and site C (*n* = 0.662) (Fig. 1C). Lastly, when we calculated the correlation of pairwise amino acid differences with the antigenic distance for previously investigated GII.4 viruses (21), the Spearman correlation analysis plot demonstrated that the variability of antigenic site A (*r* = 0.695) best describes the antigenic diversification of GII.4 noroviruses (Fig. 1D). This correlation aligns with the analysis of the temporal diversification of antigenic sites described in previous studies (6).

### Monoclonal antibodies mapped to antigenic site A demonstrate complex patterns of cross-reactivity

**Fig 2:**
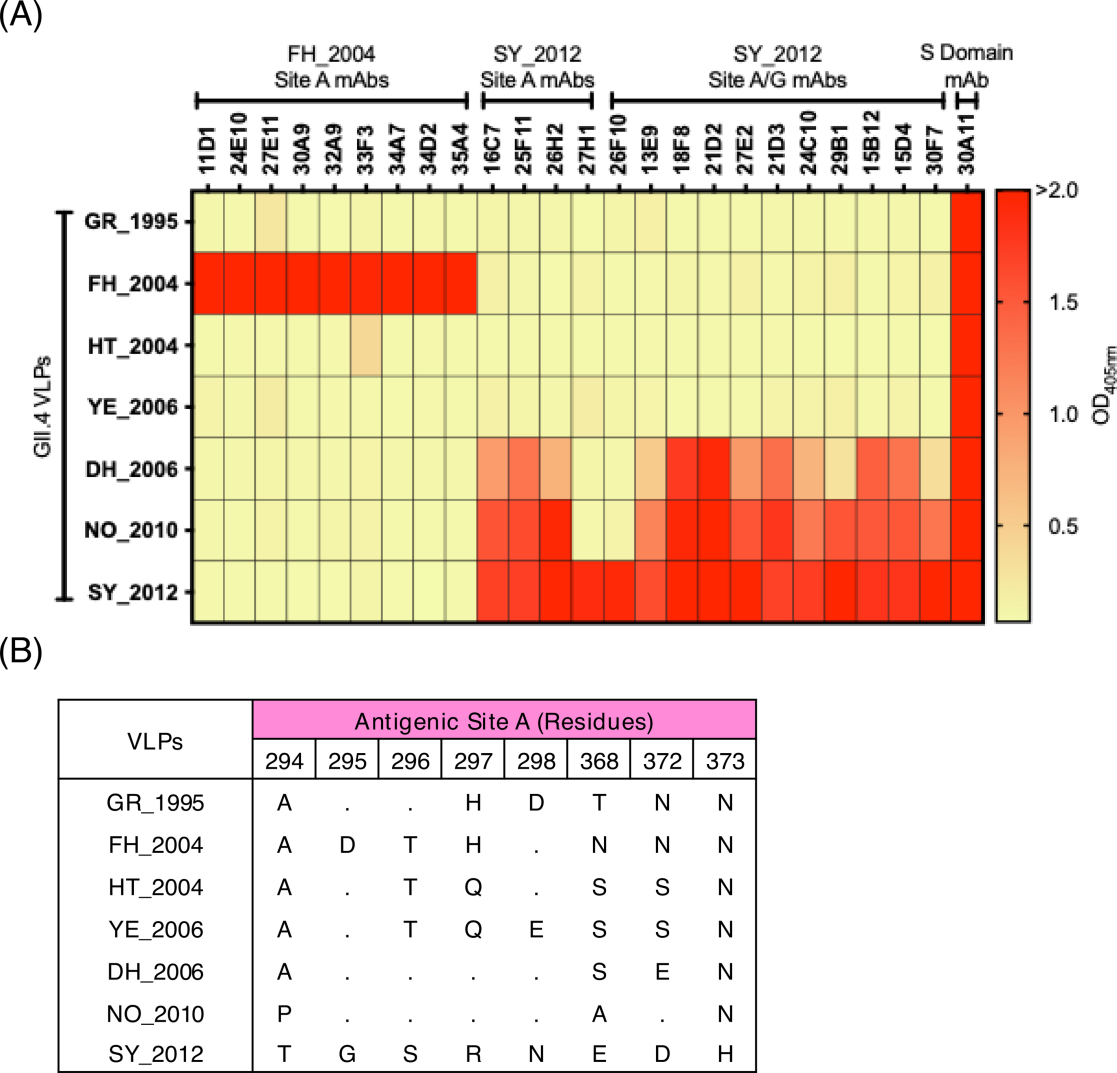
Different cross-reactivity levels of mAbs mapping to antigenic site A. (**A**) Two panels of mouse mAbs developed against FH_2004 and SY_2012 viruses that map to antigenic site A present varying levels of cross-reactivity. The variant names for each virus are summarized as follows: GR_1995, FH_2004, HT_2004, YE_2006, DH_2006, NO_2010, and SY_2012. Binding of mAbs was tested against representative VLPs from seven GII.4 variants. Each cell shows the average of the duplicate optical density at 405 nm (OD_405nm_) values at a concentration of 2 µg/mL. OD_405nm_ values of 2.00 or greater were set at the maximum, and OD_405nm_ values of 0.200 or less were set at the minimum. (**B**) Antigenic site A sequence diversity of viruses used in this study.

**Fig 3:**
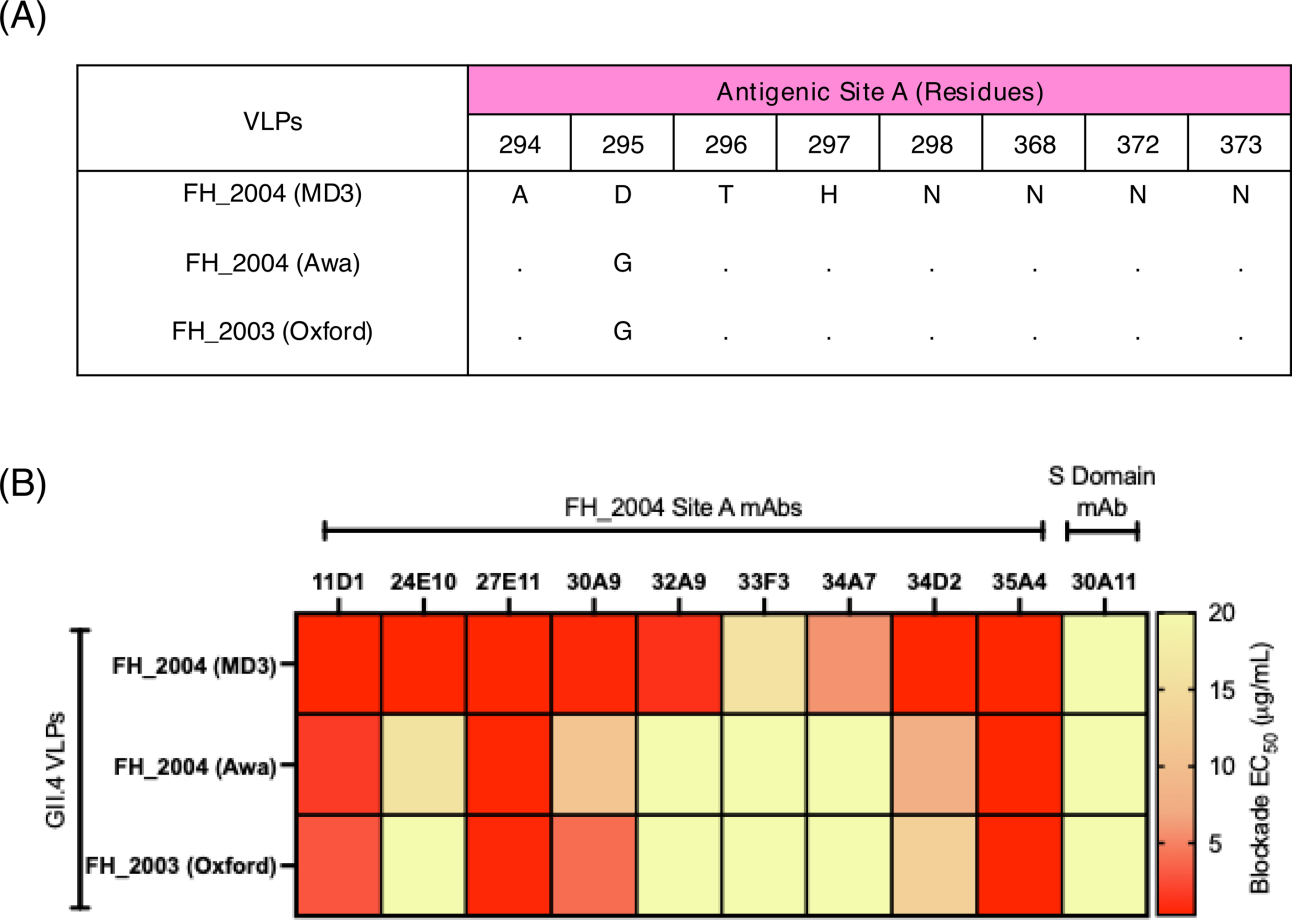
Impact of single mutation on cross-reactivity identified in Farmington Hills_2002 viruses. (**A**) Sequence alignment of antigenic site A from selected GII.4 Farmington Hills_2002 variant viruses: FH_2004 (MD3), FH_2004 (Awa), and FH_2003 (Oxford). (**B**) Heat map of the HBGA blocking of mouse mAbs elicited by antigenic site A of FH_2004 (MD3) and one shell domain mAb (30A11) to VLPs representing FH_2004 viruses. HBGA blocking of serial dilutions of mAbs was compared to the signal of the positive control, binding of VLPs in the absence of mAbs. Each cell shows the average of the 50% effective concentration (EC_50_) values calculated as the half-maximal effective concentration that results in 50% HBGA blocking of VLPs in duplicate wells.

Based on the finding that human antibodies mapping to antigenic site A can neutralize GII.4 variants circulating from 1987 to 2012 (26, 34), we sought to determine whether this could be achieved in naïve animals immunized only with single viruses. We first used two large panels of mouse mAbs developed against the Farmington Hills_2002 virus (MD3 strain detected in 2004, FH_2004; *n* = 30), and a Sydney_2012 virus (RockvilleD1 strain detected in 2012, SY_2012; *n* = 44) to test against a panel of GII.4 VLPs representing variants that emerged from 1995 to 2012 (Table 1). Of those 74 mAbs, 24 mapped to antigenic site A or antigenic sites A/G (35). The reactivity of mAbs generated against the FH_2004 VLPs showed a complete lack of cross-reactivity against any other GII.4 variant tested; however, mAbs generated against the SY_2012 VLPs demonstrated broader levels of cross-reactivity among the tested contemporary viruses (Fig. 2A; Fig. S1). As FH_2004 mAbs demonstrated a lack of cross-reactivity, we analyzed sequences presented by the other GII.4 variants tested. FH_2004 was the sole GII.4 virus to have an aspartic acid substitution at residue 295 in antigenic site A of viruses tested in this study (Fig. 2B) and all (*n* = 3,104) GII.4 virus sequences retrieved from GenBank. To measure the impact of this aspartic acid substitution, an HBGA-blocking assay was performed using all nine FH_2004 site A mAbs and two other Farmington Hills variant VLPs, FH_2004 (Awa) and FH_2003 (Oxford) (21), that do not present the aspartic acid substitution (Fig. 3A). Six of the nine mAbs either partially or completely lost blocking, highlighting the impact that a single mutation can have on blocking (Fig. 3B).

**TABLE 1:** Description of GII.4 and GII.6 norovirus VLPs used in this study

Genotype	Variant	Virus	Year	Country	GenBank accession
GII.4	GR_1995	Grimsby	1995	United Kingdom	AJ004864
GII.4	FH_2002	MD3	2004	United States	DQ658413
		Oxford	2003	United Kingdom	AY588022
		Awa	2004	Japan	AB294781
GII.4	HT_2004	Cumberland	2004	United States	EU078414
GII.4	YE_2006 a	Yerseke38	2006	Netherlands	EF126963
GII.4	DH_2006b	DenHaag89	2006	Netherlands	EF126965
GII.4	NO_2009	Virginia	2010	United States	KX353958
GII.4	SY_2012	RockvilleD1	2012	United States	KY424328
GII.6	Cluster C	Ehime	2012	Japan	AB818400

### Cross-blocking polyclonal responses attributed to antigenic site A are abundant

**Fig 4:**
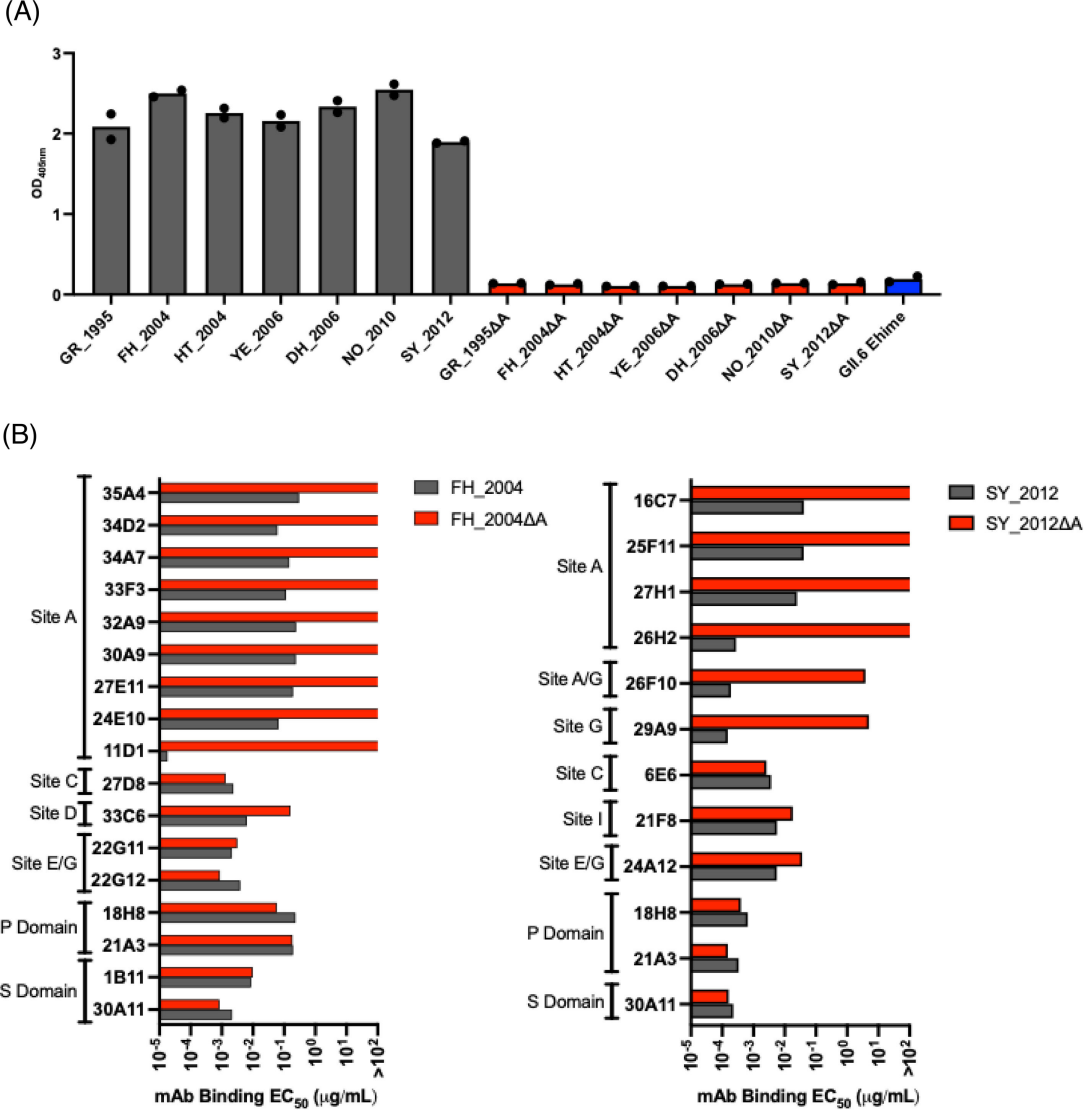
Characterization of competitors used for the HACA. (**A**) Binding of the VLPs used for the assay to HBGAs measured by the OD_405nm_ values at 2 µg/mL. (**B**) Binding of mouse mAbs from two panels, respectively, generated against FH_2004 and SY_2012. mAbs were tested on FH_2004, FH_2004ΔA, SY_2012, and SY_2012 ΔA VLPs. Each bar shows the average of the EC_50_ values calculated as the half-maximal effective concentration that results in 50% mAb binding of VLPs.

**Fig 5:**
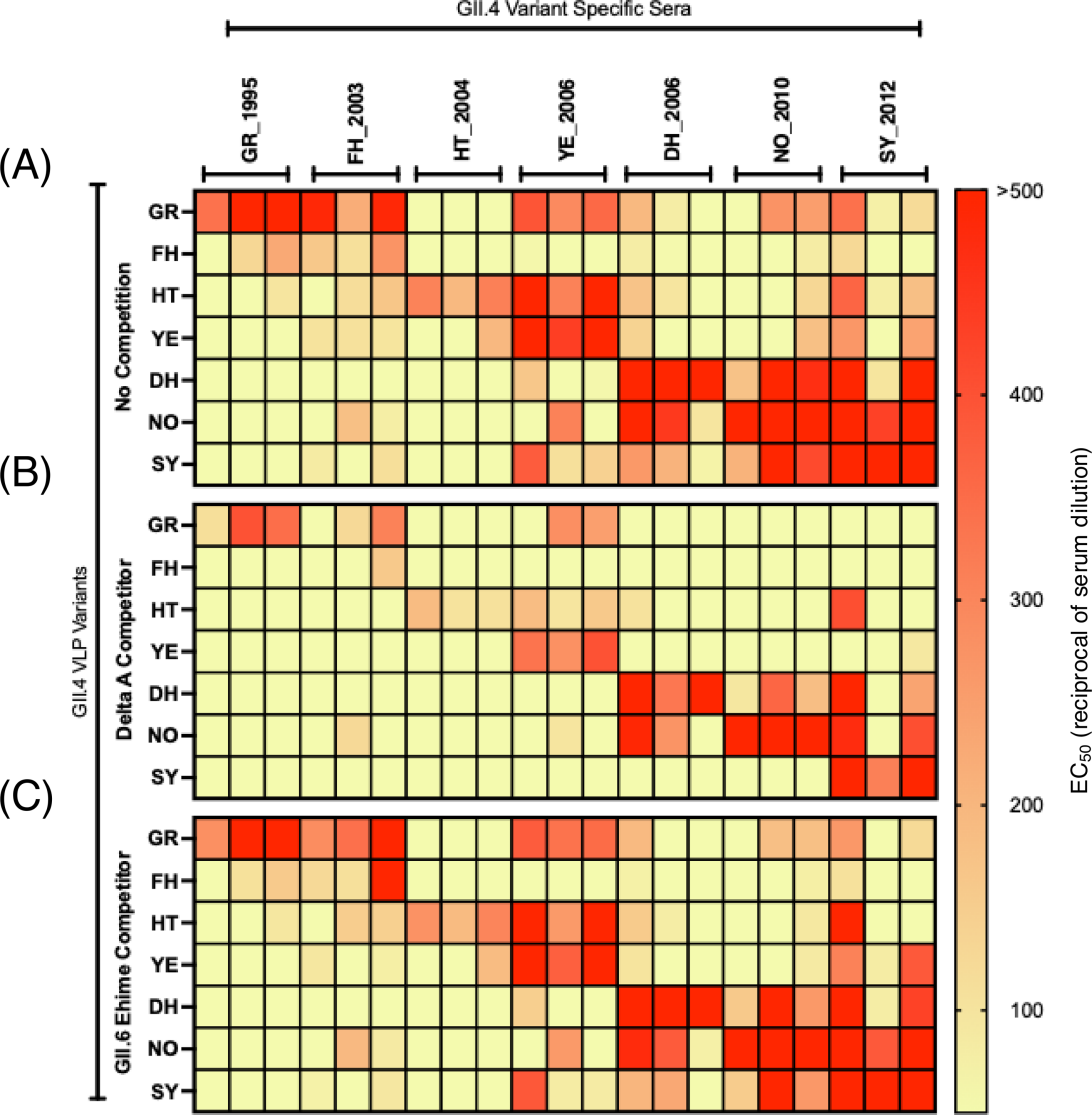
HACA from polyclonal mouse sera under different competitive conditions. (**A**) Cross-blocking of hyperimmune mouse sera (*n* = 3), generated against one of the major GII.4 viruses, was tested against seven most relevant GII.4 variants. Cross-blocking of the same hyperimmune mouse sera was competed with either a variant matched antigenic site A-depleted VLPs (**B**) or GII.6 Ehime VLPs (**C**). Each cell shows the average EC_50_ reciprocal of serum dilution values that were calculated from curves normalized to serum-only and VLP-only controls.

**Fig 6:**
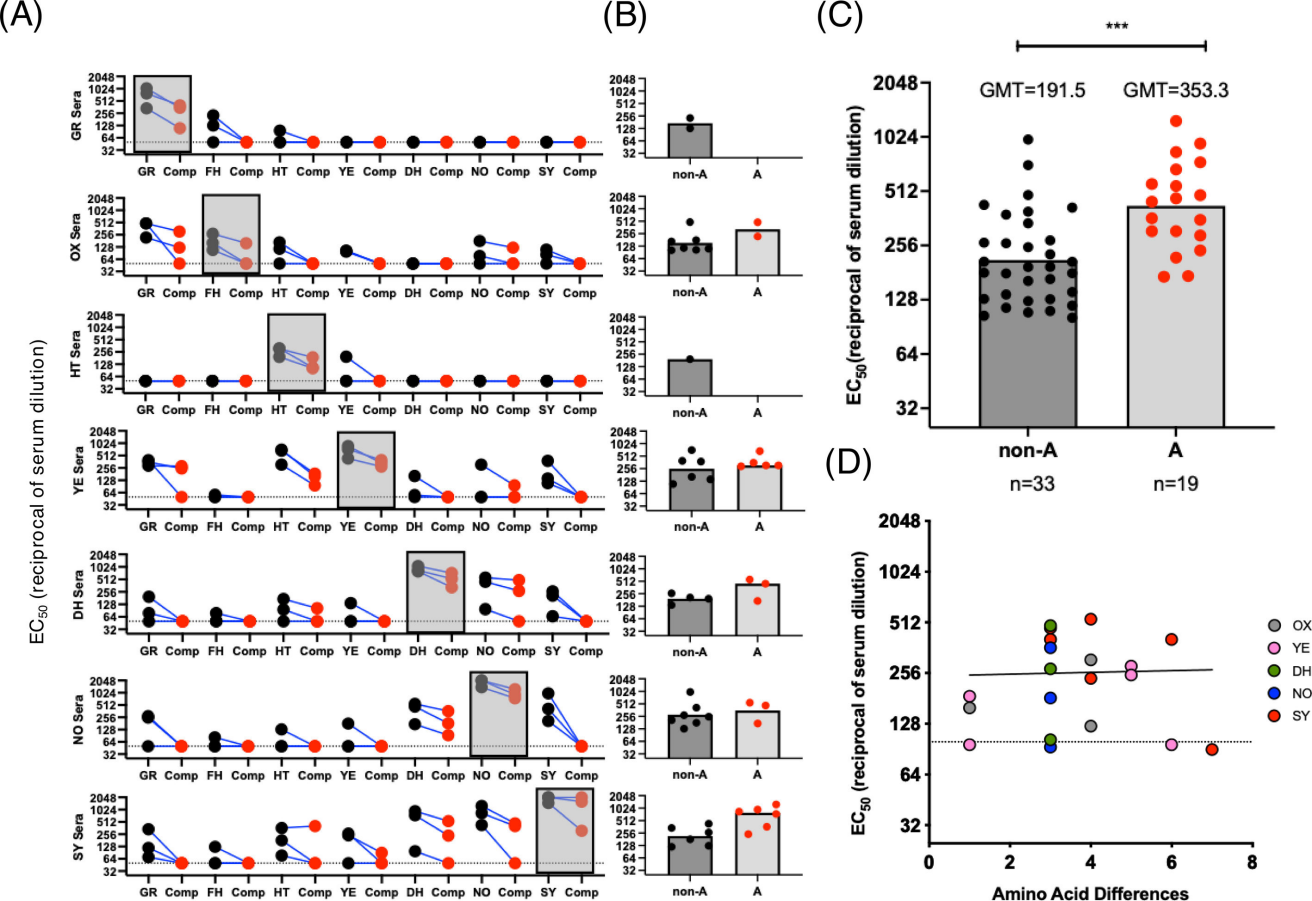
Polyclonal mouse sera demonstrate an important role of A-mapping antibodies in cross-reactivity patterns. (**A**) Line plot analysis of EC_50_ reciprocal of serum dilution HBGA blocking of hyperimmune mouse sera tested against major GII.4 variants in non-competition (black dots) and in competition with variant matched ΔA VLPs of major variants (red dots). Blue lines connecting black and red dots show the degree of antigenic site A attribution for each sera HBGA-blocking instance. Each dot represents the average EC_50_ reciprocal of serum dilution values that were calculated from curves normalized to serum-only and VLP-only controls. Dotted line is an EC_50_ limit of detection threshold. Shaded boxes highlight homologous HBGA-blocking instances. (**B**) HBGA blockade of each hyperimmune mouse sera that analyzed heterologous blocking attributed or not to antigenic site A. Heterologous HBGA blocking not associated with site A symbolized as black dots, and the EC_50_ GMT was calculated and plotted as the dark shaded bar on the left. Heterologous HBGA blocking associated with site A symbolized as red dots, and the EC_50_ GMT was calculated and plotted as a light gray bar on the right (**C**). Heterologous HBGA-blockade titers of hyperimmune mouse sera. Heterologous HBGA blocking not associated with site A presented 33 (*n* = 33) instances and symbolized as black dots, and the average GMT was calculated and plotted as the dark shaded bar on the left. Heterologous HBGA blocking associated with site A presented 19 (*n* = 19) symbolized as red dots, and the average mean titer (GMT) was calculated and plotted as a light grey bar on the right. Statistical significance was determined by unpaired *t* test of titers, ***P < 0.001. (**D**) All HBGA-blocking titers above the limit of detection for heterologous blocking were plotted along with the correlating number of mutations associated with the variant that sera blocked. Each dot symbolizes the variant-blocking sera as shown in the legend, with the solid line symbolizing the best fit of the linear regression model.

We next investigated the cross-blocking attributed to antigenic site A at the polyclonal level. We utilized a panel of hyperimmune mouse sera generated against the seven most predominant GII.4 variants (21). To test for cross-blocking while also delineating cross-blocking responses to antigenic site A, we used a novel and modified version of the HBGA-blocking assay, known as HBGA-blocking antigen competition assay (HACA) (26). The modification of the HBGA-blocking assay is that a competitor antigen protein is introduced to absorb all antibodies specific to a given site to allow for the delineation of the contribution of specific epitopes to the observed blockade. In this test, we incubated sera with wild-type (WT) VLPs of the GII.4 variants (Table 1) along with the variant matched mutant VLPs where antigenic site A has been depleted by replacing variable residues with alanine residues, named “delta A (ΔA) VLPs.” A key concept of this assay is that the competitor antigens do not bind to HBGA carbohydrates (Fig. 4A) and therefore do not modify the detection signal of the WT VLPs. We validated the antigenicity of the ΔA VLPs with mAbs mapping to different domains and antigenic sites. Only mAbs mapping to antigenic site A lost reactivity (Fig. 4B). Notably, mAb 29A9, which was mapped to antigenic site G (35), also lost binding, suggesting that this antibody recognizes an epitope encompassing antigenic sites A and G (35). Each serum was tested against each variant in a non-competition HBGA-blocking assay (Fig. 5A), a competition assay where ΔA VLPs of the corresponding variant were introduced (Fig. 5B), and a competition assay where a control, unmatched genotype GII.6 VLP, was introduced (Fig. 5C). The GII.6 Ehime VLP was selected as control because no cross-blocking was shown for GII.4 and GII.6 genotypes (26). Since the aspartic acid mutation (G295D) detected in FH_2004 (MD3) VLPs can affect the cross-reactivity, we tested the FH_2003 (Oxford) hyperimmune mouse sera as representative for the Farmington Hills variant (Fig. 5). As expected, high blockade titers were measured for each serum against the homologous VLPs, and a higher degree of cross-reactivity was measured within viruses that emerged since 2006 (Fig. 5A) (21). For example, at least seven instances of cross-reactivity were detected in mice immunized with YE_2006, DH_2006, NO_2010, or SY_2012 VLPs, but less than three instances of cross-reactivity were detected in mice immunized with HT_2004 or GR_1995 VLPs. Major differences in the HBGA-blocking titers were detected when the ΔA VLPs competitors were introduced (Fig. 5B). To quantify the cross-reactivity attributed to antigenic site A, we calculated the fold drop in HBGA-blocking titers in the competition assay as compared to the non-competition. We considered that antigenic site A-mapping antibodies contributed to cross-reactivity when less than a fourfold reduction in the HBGA-blocking titers was detected when ΔA VLPs were used as competitors in the HBGA-blocking assay. We found that in total there were 52 instances of cross-reactivity in the mouse sera (Fig. 6A), and 19 (36%) were associated with responses toward antigenic site A (Fig. 6B). No major fold differences were observed when unmatched control (GII.6) was used in the competition (Fig. S2), confirming that cross-genotype antibodies play a minimal role in HBGA-blocking activity (15, 26). Notably, nine instances of cross-reactivity were detected in mice immunized with FH_2003 (Oxford) VLPs, but only four instances of cross-reactivity were detected when using the FH_2004 (MD3) hyperimmune mouse sera (Fig. S3). Interestingly, while measuring the geometric mean titers (GMTs) for those cross-blocking 50% effective concentration (EC_50_) titers, we found that the sera elicited by GR_1995 and HT_2004 variants presented no cross-blocking titers associated with antigenic site A. In contrast, the sera elicited by contemporary viruses (YE_2006, DH_2006, NO_2010, and SY_2012) presented three or more instances of HBGA-blocking titers associated with antigenic site A (Fig. 6B). In total, antigenic site A-associated cross-blocking responses had a GMT nearly double that of those associated with other antigenic sites (Fig. 6C). Lastly, we sought to investigate the association of multiple mutations on antigenic site A with cross-blocking titers. The correlation analysis plot demonstrates that cross-blocking titers associated with antibodies mapping to antigenic site A are differentially affected by an increasing number of mutations on antigenic site A among the viruses tested (Fig. 6D); thus, some cross-reactive responses are affected by a small number of mutations while others are refractory to up to six amino acid differences. A similar pattern was demonstrated for the mAbs (Fig. S4).

## DISCUSSION

The antigenic and genetic diversity of human norovirus poses a challenge for the development of an effective vaccine. This is particularly relevant for the GII.4 genotype, which is responsible for most infections worldwide and presents the chronological emergence of predominant variants, a process similar to H3N2 influenza or severe acute respiratory syndrome coronavirus 2 (SARS-CoV-2) viruses (36, 37). Although immune responses to norovirus commonly target variable antigenic sites located at the P2 subdomain, generating variant-specific immunity, cross-neutralizing and cross-blocking responses among different variants have been described (9, 15, 20, 21, 25, 27, 29, 35, 38–40). These cross-neutralizing responses are thought to be associated with antibodies targeting conserved epitopes mapping to regions from P1 and P2 subdomains (29, 31). Robust cross-reactive responses, i.e., binding antibodies, to different norovirus strains (genotypes or variants) can be detected in previously infected individuals; however, only a small fraction of those responses will result in cross-blocking responses (26, 28). This suggests that conserved epitopes mostly elicit non-neutralizing and/or non-blocking responses (27, 29, 30), or that conserved epitopes that elicit neutralizing and/or HBGA-blocking antibodies are poorly immunogenic. A similar observation has been made for influenza viruses over a decade ago when broadly neutralizing antibodies mapping the conserved stem domain of influenza hemagglutinin (HA) were first described. Although antibodies targeting these conserved epitopes were identified (41–44), the majority of immune responses were directed toward the immunodominant and highly variable epitopes near the HA receptor-binding site (44–46), allowing viral evasion of the immune response. Despite multiple attempts to enhance the immunodominance of conserved epitopes within the HA stem domain, most efforts were unsuccessful in eliciting strong immune protective responses (41, 44). A comparable finding was observed with rationally designed norovirus VP1 antigens, in which the variable antigenic sites A and G were depleted (32).

Most neutralizing and HBGA-blocking antibody responses to GII.4 noroviruses are directed to the P domain (15), specifically to the variable antigenic sites (35). Although immunodominance of the major antigenic sites can vary from strain to strain or due to history of infections (35, 39), antigenic site A seems to be the major target for neutralizing antibody responses (23, 35). Indeed, our analyses demonstrated that antigenic site A is the most variable antigenic site and the one best explaining the antigenic diversification of GII.4 viruses. Despite the variability presented, numerous neutralizing (and/or HBGA blocking) human and mouse monoclonal antibodies presenting broad cross-reactivity to GII.4 variants were mapped to antigenic site A (20, 23, 26, 29, 34). One such antibody, NORO-123, was able to block the HBGA-binding interaction of VLPs from different GII.4 variants spanning over 25 years (34). Cross-blocking antibodies mapping to antigenic site A were also identified in polyclonal responses from individuals infected with norovirus over four decades ago (26), suggesting that strong neutralizing responses can be elicited to future emerging GII.4 strains.

In this study, we systematically evaluated the elicitation of cross-blocking antibodies mapping to antigenic site A to demonstrate extensive cross-blocking responses attributed to this site, but such responses were virus-dependent. The presence of cross-blocking antibodies mapping to antigenic site A was demonstrated in the serum from animals immunized with VLPs from noroviruses that emerged from 2006 (DH_2006, NO_2010, and SY_2012), but to a lesser extent in animals immunized with archival viruses. This reactivity pattern was previously described (21), but the extent of the role of antigenic site A in cross-reactivity was first demonstrated here. Through the use of a modified version of the HACA immunoassay, we were not only able to distinguish between antigenic site A-mapping and non-A-mapping responses but also to quantify the level of differences in their cross-blocking potencies based on varying mutations. Further analyses using a large panel of mAbs revealed the contrasting patterns of cross-reactivity associated with antibodies mapping to antigenic site A, where the SY_2012 mAbs demonstrated broader levels of cross-reactivity and cross-blocking, while FH_2004 mAbs presented a complete absence of cross-reactivity. The varying degree of impact by substitutions on antibody reactivity has been demonstrated for other viruses, like influenza virus, human immunodeficiency virus, Zika virus, foot-and-mouth disease virus, and hepatitis C virus, with single mutations causing modest to profound decreases in neutralization (47–51). Here, we showed that SY_2012 mAbs can cross-react with multiple viruses, some (e.g., DH_2006) presenting up to four substitutions at antigenic site A (Fig. 2B), while a single mutation (G295D) has been associated with the stark specificity of FH_2004 mAbs (Fig. 3). Notably, more cross-reactivity was detected in mice immunized with FH_2003 (Oxford) VLPs as compared with FH_2004 (MD3) VLPs; however, such differences in cross-reactivity were mostly associated with non-A antibodies (Fig. S3). These two viruses present one difference (G295D) in the variable antigenic sites and two additional differences (R329K and R382K) mapping outside the currently described antigenic sites. While the mutation R382K does not map on the surface, the mutation R329K is, however, surface-accessible, close to the HBGA-binding pocket, and adjacent to antigenic site G (Fig. S5) (6). The role of all these mutations on the antigenicity and cross-reactivity elicited by FH_2003 (Oxford) VLPs warrants further investigation.

A notable observation made during the development of ΔA mutant VLPs is that they do not bind HBGA carbohydrates, despite the putative-binding pocket remaining unchanged (52). A similar observation has been made for the MD145-12 virus, detected in 1987, where a single mutation, D393G, mapping to antigenic site D changed the binding profile of the virus (18). Structural and chemical changes of these positions that are neighboring to the HBGA-binding pocket can explain the differences in the HBGA-binding profile of these mutant VLPs.

Virus-dependent variations in immune responses have been identified in influenza and SARS-CoV-2, with emerging variants responding independently to contemporary variants (36, 46, 53, 54). Thus, monoclonal and polyclonal responses mapped to antigenic site A from SY_2012 were cross-reactive to the DH_2006 and NO_2010 variants; however, polyclonal responses attributed to antigenic site A from the DH_2006 and NO_2010 variants do not cross-block SY_2012 VLPs. SY_2012 VLPs demonstrated the most instances of potent cross-blocking responses associated with antigenic site A, thus making a good candidate for vaccine design. Humans have been shown to be infected with norovirus several times throughout their lifetime (55), and primary GII.4 infection heavily influences their secondary responses to different GII.4 variants (39). Thus, a limitation of this study was the availability of sera samples from individuals whose primary infection was linked to different GII.4 viruses to assess whether the results presented here will translate to human responses.

Overall, our study demonstrates the complex cross-reactive pattern elicited by the most immunodominant and variable antigenic site A, and though further studies are required to better understand the interaction of viral antigenic evolution and elicitation of potent antibodies resistant to viral escape, these data could provide helpful insights toward the development of cross-protective vaccines against human norovirus.

## MATERIALS AND METHODS

### Bioinformatics analyses

A total of 3,104 full-length VP1 sequences of the GII.4 genotype were downloaded from GenBank and analyzed with an internal R script, which is available upon request (6, 25). Entropy values were calculated for each residue comprising each antigenic site utilizing the Entropy-One tool, as implemented in Los Alamos National Laboratory (https://www.hiv.lanl.gov/content/sequence/ENTROPY/entropy_one.html). GII.4 capsid variant distribution and their respective sequence pattern for each of the five antigenic sites were plotted using the R script output, Entropy-One tool output, and GraphPad Prism (10.2.3). Structural rendering of the VP1 protein with the different antigenic sites was done using ChimeraX (56) and the X-ray model of the SY_2012 virus (PDB: 4OP7). Correlation plot analysis was done by pair-wise analyses where the *x*-axis was the amino acid differences at antigenic site A and the *y*-axis was the EC_50_ value between the variant used for immunization against the EC_50_ value of heterotypic responses, followed by a linear regression function as implemented in GraphPad Prism (10.2.3).

### Virus-like particles and antibodies

The Bac-to-Bac Baculovirus expression system was utilized to generate WT and mutant VLPs (Table 1) (35). Briefly, VP1 sequences were obtained from GenBank, synthesized, and then cloned into the pFastBac1 plasmid. These pFastBac1 plasmids were then transformed into MAX efficiency DH10Bac competent *Escherichia coli* cells (Thermo Fisher Scientific) to obtain the bacmids. For ΔA VLPs, the VP1 sequences were synthesized with residues from antigenic site A replaced with alanines. Transfection of Sf9 insect cells (ATCC Catalog # CRL-1711) with the purified bacmids was done with Cellfectin II (Gibco 10362-100). Baculovirus recovery was confirmed by cell viability and expression of norovirus VP1. Norovirus VLPs were purified with ultracentrifugation, and their structural integrity was confirmed by negative stain electron microscopy. Hyperimmune sera against WT VLPs were produced in BALB/c mice by intramuscular immunization with 100 µL of VLP (0.4 mg/mL) with alum (Alhydrogel adjuvant 2%, Croda) and two boosts on weeks 4 and 8. Terminal bleed was performed on week 10. All the VLPs, hyperimmune mouse sera, and mouse mAbs were described previously (21, 35).

### Enzyme-linked immunosorbent assay

Antibody reactivity profiles were generated by testing mouse mAbs against the VLPs through enzyme-linked immunosorbent assay (ELISA) as described previously (6). Briefly, a 96-well U bottom plate that was coated with 0.5 µg/mL of VLPs in 1× phosphate buffered saline (PBS) overnight at 4°C. Wells were then washed in a wash buffer consisting of 1× PBS containing 0.1% Tween 20, followed by blocking for 1 hour at room temperature with 1× PBS with 5% blocking buffer. In duplicate, twofold serial dilutions (starting at 2 µg/mL) of mouse mAbs were incubated for 1 hour at 37°C. Plates were washed three times using wash buffer, and 1:2,000 anti-mouse IgG conjugated with horseradish peroxidase (HRP) in 5% blocking buffer was incubated for 1 hour at 37°C. ABTS 1-Component Microwell Peroxidase Substrate (SeraCare) was then incubated in wells and quantified as an optical density at 405 nm (OD_405nm_) using the Spectrostar nano plate reader. The EC_50_ values were then calculated using parametric analysis as implemented in GraphPad Prism (10.2.3).

### HBGA-blocking assay

The assay that measures the VLPs binding to HBGAs and antibody-mediated blockade of VLP binding to HBGAs was described previously (27). The HBGA-blocking assay is briefly described here: 96-well U bottom polyvinyl plates were coated with pig gastric mucin III (PGM-III) at a concentration of 10 µg/mL after overnight incubation at 4°C. A separate 96-well U bottom plate was incubated with 5% blocking buffer overnight at 4°C. The plate with blocking buffer was then washed and incubated with twofold serial dilutions of mAbs (starting dilution: 20 µg/mL) or hyperimmune mouse sera (starting dilution: 1/50). VLPs at a concentration of 0.5 µg/mL were added to the wells and incubated for 1 hour at 37°C. The plate containing PGM was washed and incubated at room temperature for 1 hour with 5% blocking buffer. PGM plate with blocking buffer was then washed, and wells incubating antibody and VLP were transferred to a plate coated with PGM. PGM-coated plate with antibody and VLPs wells were then incubated at 37°C for 1 hour. The plate was washed in wash buffer four times, and a 1:5,000 anti-GII.4 guinea pig was incubated for 1 hour at 37°C. Wells were then washed four times with wash buffer, and a detection anti-guinea pig IgG labeled HRP (SeraCare) at 1:2,000 was added and incubated in wells for 1 hour at 37°C. Lastly, wells were washed again four times in wash buffer, and ABTS 1-Component Microwell Peroxidase Substrate (SeraCare) was added, and absorbance was measured at OD_405nm_. EC_50_ was calculated as described in “Enzyme-linked immunosorbent assay,” above. The association between EC_50_ and amino acid differences was analyzed using GraphPad Prism (10.2.3).

### HACA

This assay was recently described in Pilewski et al. (26). Briefly, the setup for this assay is as described for the HBGA-blocking assay. The modification occurs when mouse sera samples are incubated with the testing WT VLPs (0.5 µg/mL). In this step, the competitor antigenic site A-depleted VLPs (ΔA VLPs) or GII.6 Ehime (negative control) are added to wells at a concentration of 5 µg/mL. The non-competition well received 5% blocking buffer. The following steps occur as described in the HBGA-blocking assay with bound VLPs detected with anti-GII.4 guinea pig sera. The attribution of antigenic site A and cross-reactivity were normalized using the GII.4 variant corresponding to the WT non-competition. EC_50_ and GMTs were calculated using GraphPad Prism version (10.2.3).

## Data Availability

The sequences used in this study were downloaded from the GenBank public database and are available upon request. The viral sequences used to develop the VLPs are available in Table 1. VLPs, sera, and mAbs used in this study are available upon request.

## SUPPLEMENTAL MATERIAL

Figure S1 jvi.00652-25-s0001.tiff Normalized OD curves in ELISA binding assays of six site A SY_2012 mAbs.

Figure S2 jvi.00652-25-s0002.tiff Cross-reactivity patterns of polyclonal mouse sera are not affected by competition with unrelated genotype.

Figure S3 jvi.00652-25-s0003.tiff Comparison of cross-reactivity patterns of polyclonal sera from mouse immunized with two FH_2004 viruses.

Figure S4 jvi.00652-25-s0004.tiff Effect of varying levels of mutations on cross-reactivity in mouse mAbs generated against a SY_2012 virus.

Figure S5 jvi.00652-25-s0005.tiff GII.4 norovirus structural model of GII.4 P domain highlighting the antigenic sites A, C, D, E, and G.

ASM does not own the copyrights to Supplemental Material that may be linked to, or accessed through, an article. The authors have granted ASM a non-exclusive, world-wide license to publish the Supplemental Material files. Please contact the corresponding author directly for reuse.
