# Supplementary figures and images for: Highly variable antigenic site located at the apex of GII.4 norovirus capsid protein induces cross-reactive blocking antibodies in a variant-specific manner
Source: J Virol. 2025 May 30;99(7):e00652-25. doi: 10.1128/jvi.00652-25 (PMC12282187; doi:10.1128/jvi.00652-25)

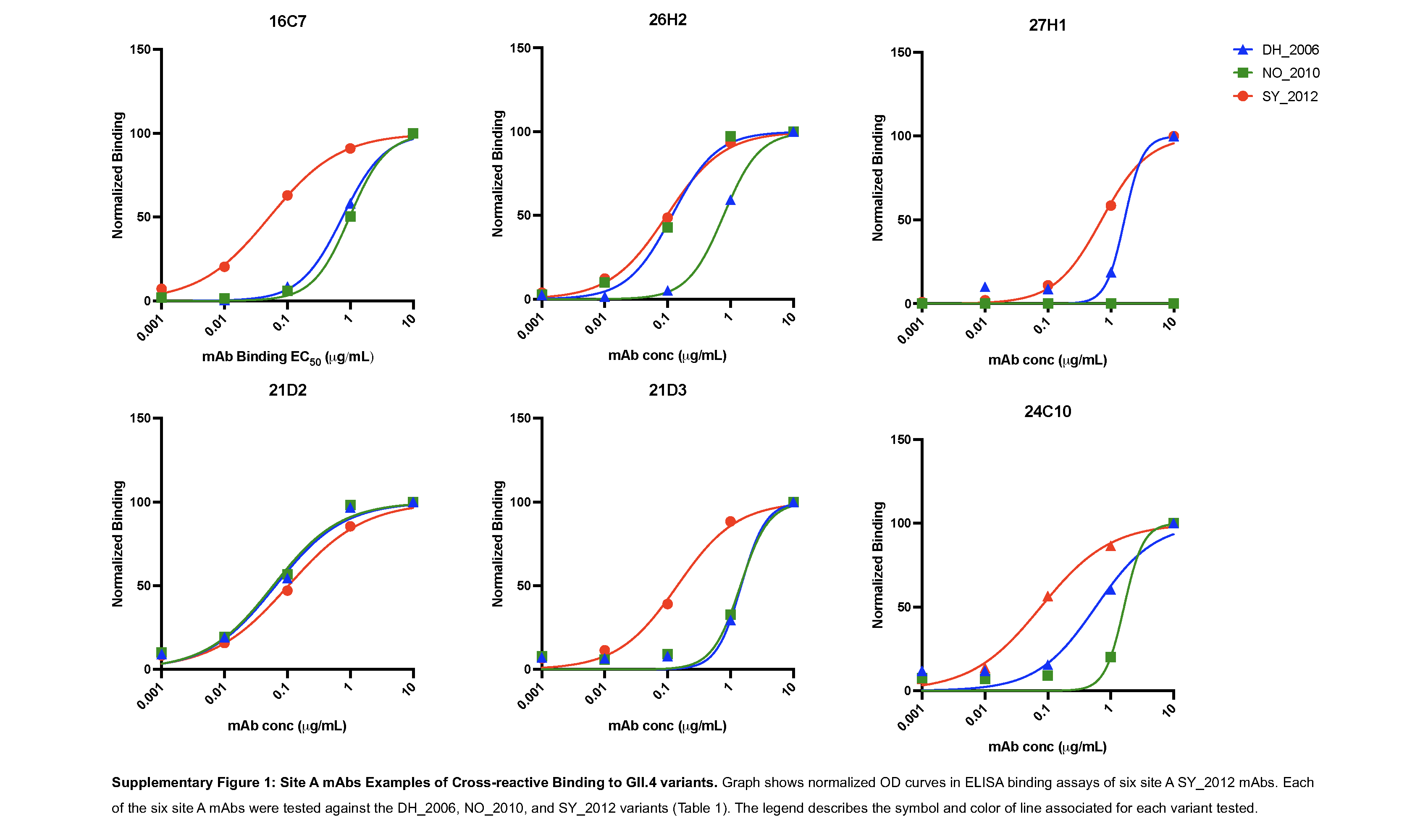

Supplement: Figure S1 — Normalized OD curves in ELISA binding assays of six site A SY_2012 mAbs. [file jvi.00652-25-s0001.tiff]

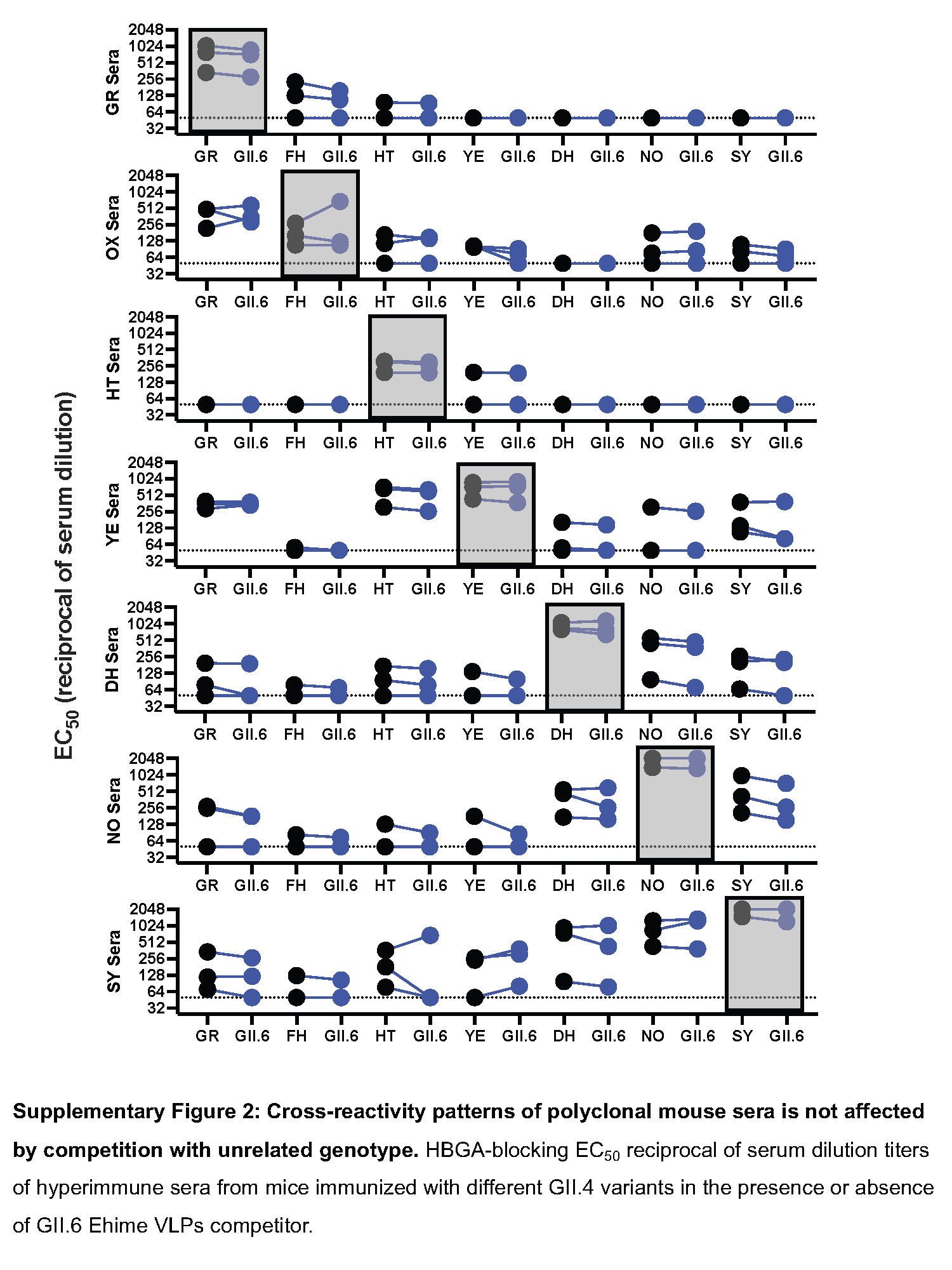

Supplement: Figure S2 — Cross-reactivity patterns of polyclonal mouse sera are not affected by competition with unrelated genotype. [file jvi.00652-25-s0002.tiff]

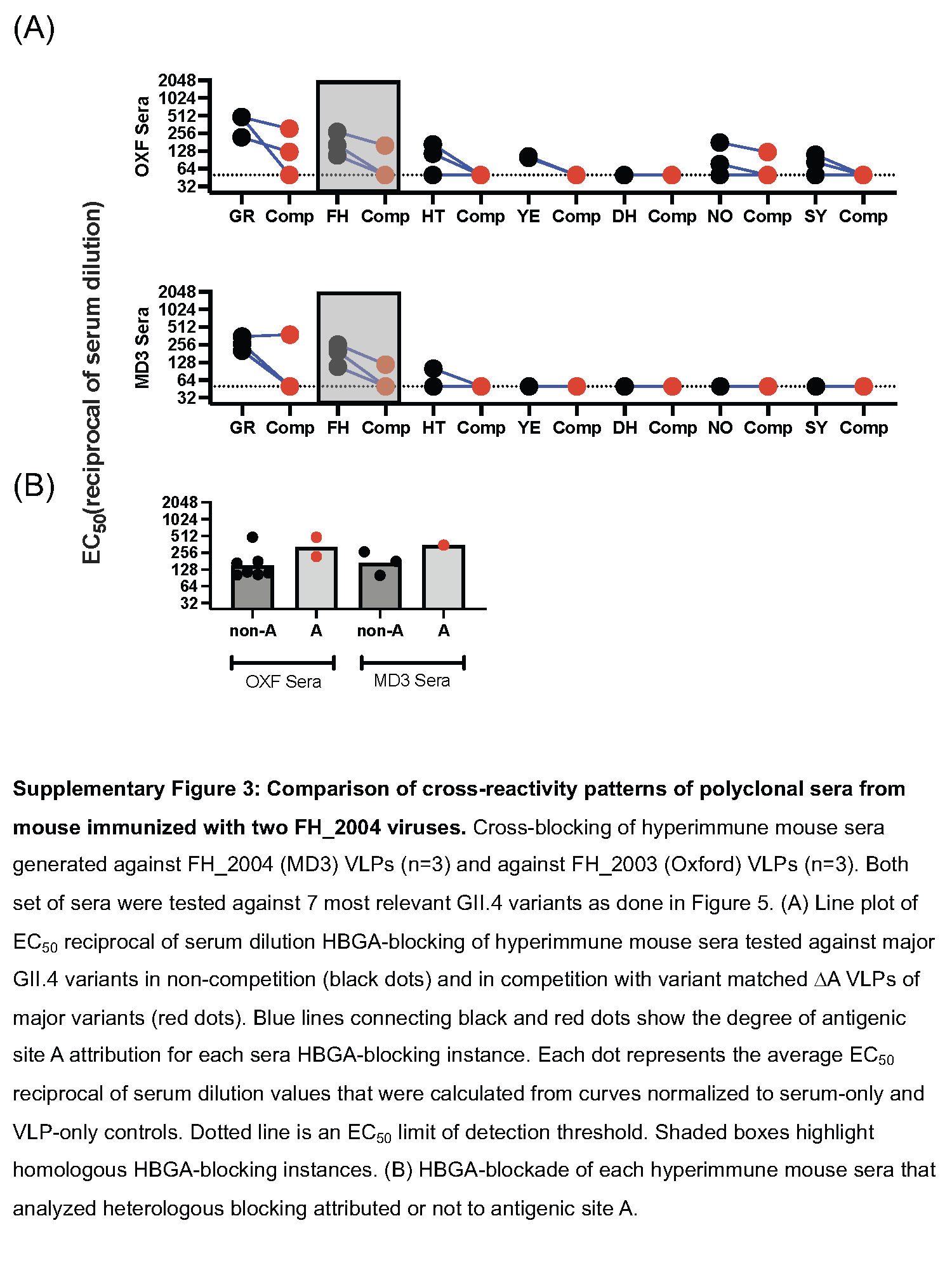

Supplement: Figure S3 — Comparison of cross-reactivity patterns of polyclonal sera from mouse immunized with two FH_2004 viruses. [file jvi.00652-25-s0003.tiff]

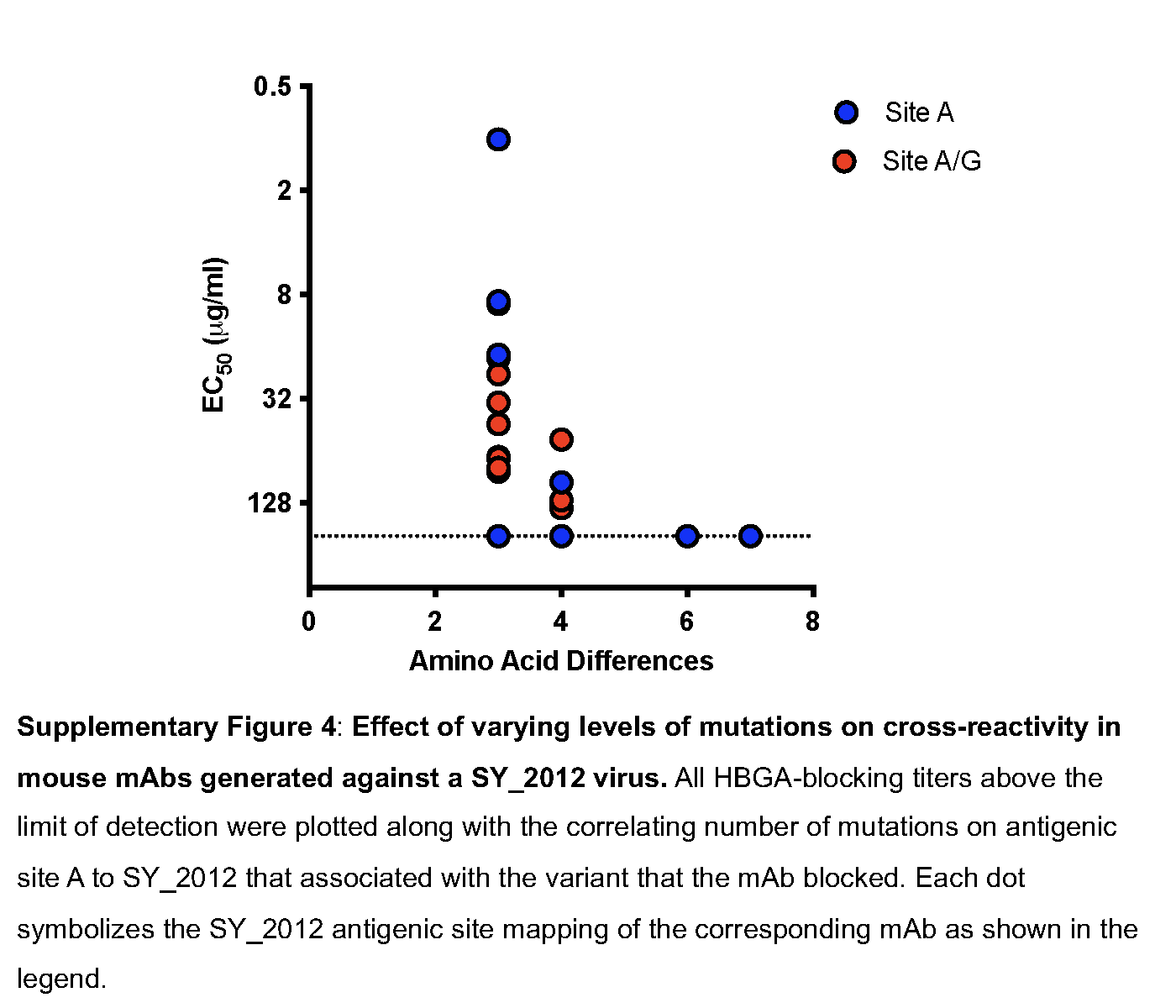

Supplement: Figure S4 — Effect of varying levels of mutations on cross-reactivity in mouse mAbs generated against a SY_2012 virus. [file jvi.00652-25-s0004.tiff]

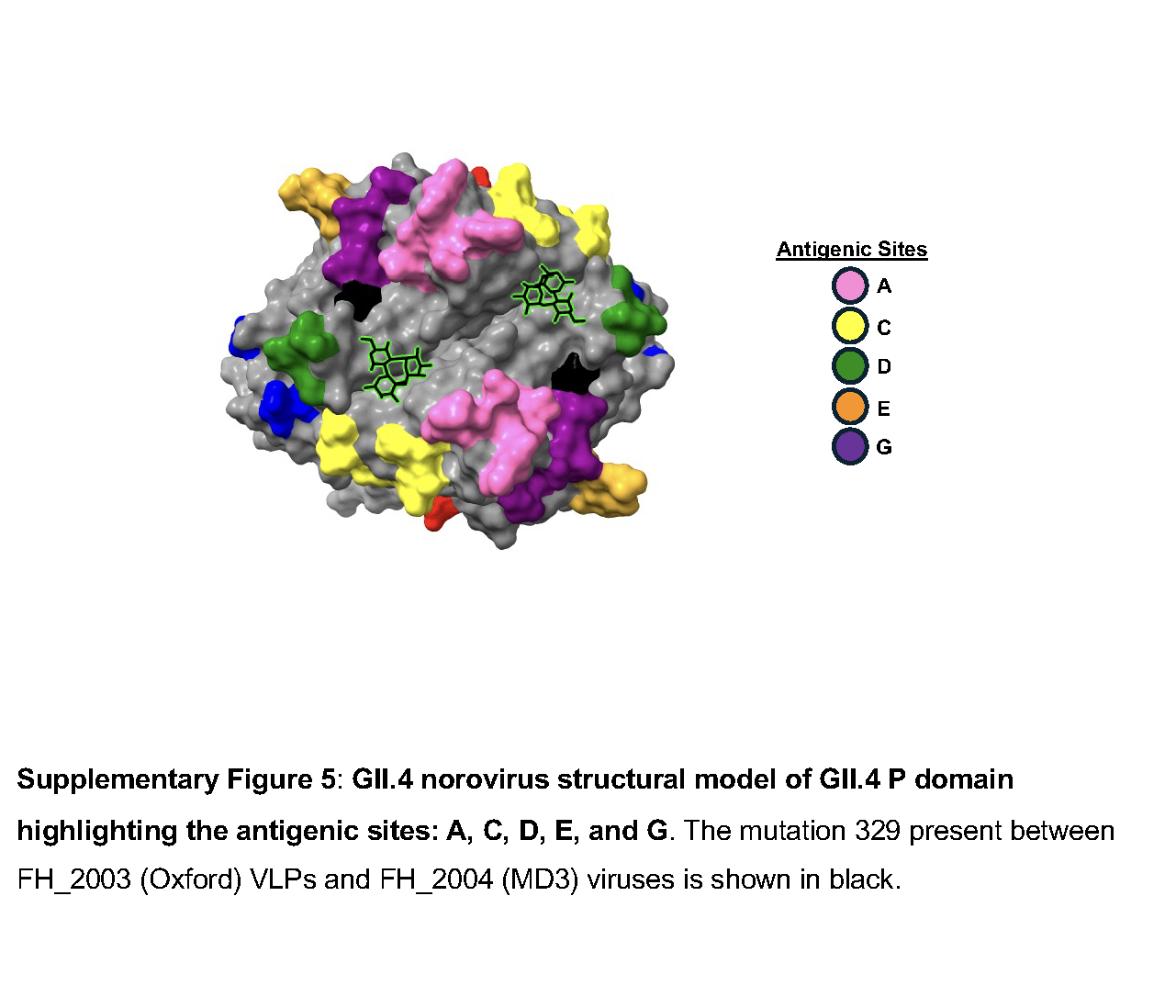

Supplement: Figure S5 — GII.4 norovirus structural model of GII.4 P domain highlighting the antigenic sites A, C, D, E, and G. [file jvi.00652-25-s0005.tiff]
